# Scaling Up Malaria Control in Zambia: Progress and Impact 2005–2008

**DOI:** 10.4269/ajtmh.2011.10-0617a

**Published:** 2011-02-04

**Authors:** I. M. Spence-Lewis

**Affiliations:** Fellow, Faculty Development Program; National Center for Primary Care; Morehouse School of Medicine; Atlanta, GA 30310; E-mail: drspencelewis@aol.com

Dear Sir:

Your publication of the assessment of Zambia's National Malaria Control Program by Elizabeth Chizema-Kaweshia and others^1^ documents successful outcomes using evidence-based Integrated Vector Management in Zambia. World Health Organization (WHO) recommendations and goals of the Global Roll Back Malaria Partnership contributed a proven framework for implementing Zambia's national malaria control strategies. There is agreement regarding Integrated Vector Management as an important strategy for the national prevention and control of malaria in Zambia and other African Countries with endemic malaria.^2^ An integrated approach to vector management is critical to maintaining and improving Zambia's extensive collaborative efforts with multilateral and bilateral agencies and stakeholders globally and nationally.

Between 2005 and 2008, Zambia applied Integrated Vector Management to malaria prevention and control. The results showed measurable improvements in the successful prevention and advancement of malaria control in Zambia. However, the authors did not acknowledge achievements and compare lessons learned from other African countries recording similar success with malaria control programs. There is a lack of data regarding capacity building for health communication among indigenous people in Zambia.^3^ Health communication tools and strategies in Zambia's Strategic Plan would support advocacy for wider rural community participation, coverage, and leadership.
